# Niemann–Pick type C disease – the tip of the iceberg? A review of neuropsychiatric presentation, diagnosis and treatment

**DOI:** 10.1192/pb.bp.116.054072

**Published:** 2017-04

**Authors:** William R. H. Evans, Chris J. Hendriksz

**Affiliations:** 1 Niemann-Pick UK, Washington, UK; 2 The Mark Holland Metabolic Unit, Salford Royal Foundation NHS Trust, Manchester, UK

## Abstract

Niemann–Pick type C (NP-C) disease is a rare neurodegenerative lysosomal storage disorder. It is highly heterogeneous, and there is limited awareness of a substantial subgroup that has an attenuated adolescent/adult-onset disease. In these patients psychiatric features, often a psychosis, may dominate the initial impression, although often there is an associated ataxia and cognitive impairment. Typically, patients experience a substantial diagnostic delay. In this review we highlight the importance of early recognition and discuss the pathophysiology, neuropsychiatric presentation and recent changes in the investigation and work-up of these patients, and treatment options.

Inborn errors of metabolism (IEM) are a collection of diseases that result from a deficiency in a metabolic pathway (usually an enzyme), leading to altered intracellular synthesis and catabolism.^1^ IEMs are individually rare but collectively common. Most are diagnosed during childhood, but there is increasing awareness of later-onset variant forms that may present in adults, often with a mix of both cognitive and psychiatric problems.^2^

Niemann–Pick type C (NP-C) disease is a neurodegenerative, pan-ethnic, globally occurring lysosomal storage disorder. Lysosomal storage disorders are a subgroup of nearly 60 IEMs that also includes Gaucher's disease, Tay–Sachs disease and the mucopolysaccharidoses. Although the lysosomal storage disorders are individually rare, their collective prevalence is 1:5000;^3^ they are usually the consequence of an enzyme deficiency and follow an autosomal pattern of inheritance. NP-C is rare, with a ‘classical’ clinical incidence of approximately 1:100 000.^4–7^ It follows an autosomal inheritance pattern, the result of mutations in one of two genes: *NPC1* (chromosome 18q 11-12) or *NPC2* (chromosome 14q 24.3),^8^ with *NPC1* accounting for 95% of cases.^4,6^ However, unusually, neither of these genes encodes an enzyme; they encode intracellular transporter proteins: *NPC1*, a late endosome/lysosomal transmembrane-bound protein and *NPC2*, a soluble protein.^9^

NP-C, like many other IEMs, has historically been considered a severe neurological and systemic disease of children, but this does not reflect the wide range of its presentations and severities. In a substantial subgroup of patients the illness has an adolescent/adult onset, with cognitive decline and neuropsychiatric symptoms predominating and survival that can extend even into their 7th decade.^10^ Most of these patients never receive an accurate diagnosis and for those who do, it is often after many years' delay with frequent misdiagnoses.^1,4,10–12^

This is a timely review of adult- and adolescent-onset NP-C. A recent publication suggests that the frequency of this late-onset disease may be far higher than the 1:100 000 ‘classical’ incidence.^4–7,13^ Wassif *et al*^6^ predicted a prevalence as high as 1:19 000–36 000, based on exome sequencing data of known disease-causing mutations. With easier and more readily available diagnostic tests, a disease-specific treatment^4^ and ongoing clinical trials, there has never been a more important time for a heightened awareness of NP-C.

## Pathophysiology

The lysosome is an intracellular organelle, often termed the recycling centre of the cell. It has an acidic interior containing hydrolytic enzymes (hydrolases). These hydrolases, together with the integral transporter proteins (such as NPC1 and NPC2), traffic, break down and recycle cellular products. A defect in these results in the accumulation of partially metabolised substrates and a shortage of other lysosomal products. This ‘traffic jam’ leads to a complex chain of events, resulting in cell dysfunction and death and the consequent disease phenotype.

NP-C was described more than 100 years ago by Albert Niemann as an infantile disorder with hepatosplenomegaly and neurodegeneration,^9^ but the exact function of the NPC1 and NPC2 proteins has still to be fully elucidated. We know that a loss of function of either results in an identical clinical phenotype, suggesting a shared pathway for the two proteins. NPC1, a large transmembrane protein of the late endosome/lysosome and NPC2, a soluble lysosomal protein,^8^ work cooperatively to traffic intracellular lipids. Loss of function in either protein leads to the accumulation of cholesterol and a range of sphingolipids in the late endosomal/lysosomal intracellular compartment. This disrupts lysosomal calcium homeostasis, resulting in a host of secondary cellular trafficking defects.^14^ The neuropathological sequelae of these defects include Alzheimer's-like neurofibrillary tangles, neuronal degeneration, neuroaxonal dystrophy and demyelination.^6–18^ Also, as endogenously synthesised cholesterol is necessary for axonal membrane maintenance and repair, white matter tracts are severely affected, with the corpus callosum showing the most striking axonal loss.^18,19^ Purkinje cells of the cerebellum, basal ganglia and thalamus are characteristically vulnerable in NP-C, leading to the often pronounced cerebellar dysfunction and ataxia in NP-C patients.^15^

## Neuropsychiatric presentation

An organic cause can be found in a sizeable proportion of patients presenting with psychosis. For example, Johnstone *et al*^20^ showed that a causative organic disease could be found in 6% of patients with a first episode of psychosis. Several IEMs are known to cause both psychoses and cognitive decline in adults. A recent systematic review^2^ highlighted six metabolic disorders that should be considered in adult patients with psychosis: homocysteine metabolism disorders, urea cycle disorders, Wilson's disease, acute porphyrias, cerebrotendinous xanthomatosis and NP-C.

NP-C can vary widely in both age at onset and symptoms. A useful classification system subdivides NP-C into four groups based on the onset of neurological disease:

- early infantile
- late infantile
- juvenile
- adolescent/adult onset.^4^

**Fig. 1:**
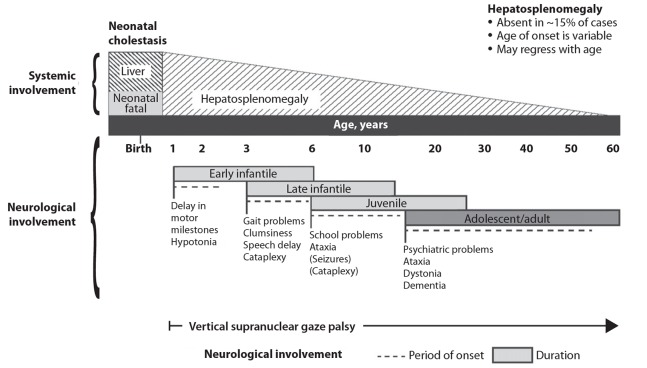
Visceral and neurological manifestations in Niemann–Pick type C disease. Reprinted from Patterson *et al*,^4^ copyright 2012, with permission from Elsevier.

Typically, the earlier the onset of neurological disease, the more aggressive the disease process (Fig. 1).^4,21,22^

Patients with adolescent/adult-onset NP-C have a neuropsychiatric disease involving varying degrees of cognitive decline, psychiatric and neurological symptoms.^4^ At presentation, psychiatric symptoms often dominate the clinical impression, and a substantial period may pass before neurological symptoms develop or are recognised by treating clinicians.^22^ In many patients this delay is confounded by early neurological features, commonly an ataxia misinterpreted as a side-effect of psychotropic medication, and the challenge of eliciting subtle cognitive decline in a depressed or psychotic patient.^22,24^ This diagnostic delay is often measured in years or sometimes even decades,^10,24^ with patients frequently receiving a range of incorrect neurological and psychiatric diagnoses before NP-C is confirmed. Incorrect diagnoses made in patients with NP-C include:^12^

- psychotic syndromes
- Alzheimer's disease and frontotemporal dementia
- progressive supranuclear palsy
- Parkinson's disease/parkinsonism
- spinocerebellar ataxia
- Wilson's disease
- multiple sclerosis
- Creutzfeldt–Jakob disease
- Wernicke encephalopathy.

### Cognitive decline

The neurodegenerative disease leads to dementia in almost all NP-C patients. NP-C is sometimes referred to as ‘childhood Alzheimer's’. Although this is used as an easy identifier rather than for its accuracy, the two diseases share some neuropathological features.

In patients with adult/adolescent onset, cognitive decline features to a greater or lesser extent in almost all cases. If found in combination with other disease features, further investigations should be performed.^25^ To highlight a possible diagnosis, the three most useful clinical features are: cognitive decline, psychosis and progressive ataxia. Consequently, these have been utilised in a patient group-initiated awareness campaign ‘Think again, think NPC’ (http://think-npc.com). This clustering of symptoms to help identify patients has been further investigated by Wijburg *et al*^25^ who have developed ‘The NP-C Suspicion Index’. This index attributes scores to different clinical features to identify the likelihood of a patient having NP-C, with pre-senile cognitive decline (< 40 years) considered a strong indicator.^25^

The cognitive profile in adult patients with NP-C usually starts with problems in word fluidity, working memory and executive dysfunction.^4,8,26^ There may also be a frontal lobe syndrome with perseveration and loss of interpersonal distance that manifests as excessive familiarity.^26^ At this early stage of cognitive impairment, the Mini-Mental State Examination (MMSE) often fails to identify deficiencies in these areas. However, with disease progression, a more global impairment of function develops, meeting the diagnostic criteria for dementia.^4,5,21,22^

### Psychiatric symptoms

Psychiatric symptoms associated with NP-C can vary. In juvenile- and adolescent-onset patients, intellectual disability, behavioural problems and attention-deficit hyperactivity disorder (ADHD) have been reported.^4,11,22^ Many of these patients receive further psychiatric diagnoses at a later stage.^4^ One case report describes a patient with an autism spectrum-like disorder preceding a schizophrenia-like illness before eventually receiving their final diagnosis of NP-C.^27^

In adult patients both affective disorders and psychoses are reported.^4,5,22^ An NP-C case series describes general psychiatric symptoms in 38% of early adult-onset patients,^22^ with the literature suggesting that a schizophrenia-like disorder is found in 25–40% of adolescent- and adult-onset NP-C patients.^4,22^ This psychiatric disease may initially be indistinguishable from schizophrenia, with auditory hallucinations, delusions and disorders of thought and behaviour; however, certain features are suggestive of an organic cause:

- neurological or visceral features
- cognitive impairment
- treatment resistance or even a paradoxical worsening of psychosis with drug therapy
- visual hallucinations, unusual in classical forms of schizophrenia.^4^

Although the psychosis may be resistant to therapy, atypical antipsychotics can be useful, but caution should be taken to avoid worsening of any pre-existing dystonia.^4,18^

Other major psychiatric illnesses described in NP-C include:

- depression generally susceptible to selective serotonin reuptake inhibitor (SSRI) therapy^4^
- bipolar disorder, often sensitive to mood stabilisers such as sodium valproate^4,18^
- obsessive–compulsive behaviour
- catatonia, often in younger patients and sometimes resistant to treatment, although electroconvulsive therapy (ECT) has been used successfully.^4,18^

### Neurological features

Adolescent- and adult-onset NP-C patients almost always have some neurological features at presentation, although these may at first be subtle and eclipsed by psychiatric features. In the more aggressive late infantile/juvenile-onset group, patients are often first described as being clumsy and struggling at school. This then progresses to the development of frank neurological disease that may include limb and gait ataxia, seizures, gelastic cataplexy (the loss of muscle tone with emotional stimuli), dysarthria, dystonia, dysphagia and dementia. Prognosis in these patients is poor, with death from the consequences of their advanced neurological disease typically in their late teenage years or early adulthood.^4,7^

Adolescent and adult patients share some of these disease features, but in their case the illness is more insidious in its onset and slower in progression. Cerebellar dysfunction, especially ataxia, is the most commonly identified neurological feature, although dysarthria and dystonia are also frequently present.^4,18,22^ Interestingly, epilepsy, common in infantile and juvenile disease, and cataplexy (20% of classical NP-C patients), are both rarely seen.^22^

The most important neurological sign in NP-C, as it is both highly prevalent and specific, is a vertical supranuclear gaze palsy (VSGP).^4,7^ VSGP is seen in only a limited number of other neurodegenerative diseases and rarely so early in their disease process. In NP-C it nearly always heralds the onset of the neuropsychiatric disease, regardless of the patient's age.^4,22^

The gaze palsy, initially in the vertical plane, progresses to also involve horizontal eye movements as the brainstem pathology advances. Initially, the VSGP is subtle and may be missed. It involves vertical voluntary saccadic movements only, especially of downward gaze, and at this stage slow pursuit eye movements are preserved.^4^ If saccadic eye movements are not tested, the initial VSGP will be missed. Saccadic eye movements are easily tested by requesting the patient to look up and then down in quick repetition. (See http://think-npc.com/could-it-be-np-c for a video demonstrating saccadic eye movement testing in NP-C.)

### Systemic features

NP-C is a neurovisceral disease, but in adolescents and adults the visceral component is rarely of clinical significance, although splenomegaly with or without hepatomegaly is usually present.^4^

In the perinatal and early juvenile forms, systemic manifestations may be pronounced, with severe and sometimes fatal liver and pulmonary disease.^4^ Interestingly, regardless of the patient's age, visceral disease, when present, always precedes neuropsychiatric features, often by years or even decades. The severity of this visceral disease offers little insight into the likely onset or severity of the patient's later neurological disease.^4,5^ There are cases of patients with paediatric liver disease who only develop neuropsychiatric features many decades later in adulthood.^12^

In adolescent- and adult-onset patients, hepatosplenomegaly – although frequently present – is often unrecognised. When present it is usually less pronounced and nearly always asymptomatic.^4^ The proportion of patients with hepatosplenomegaly in one case series was 85%, but within the adolescent/adult cohort it was reported lower, at nearer to 50%.^5^ However, another group reported that splenomegaly (with or without hepatomegaly) was found on abdominal ultrasound in closer to 90% of patients, regardless of the patient's age.^4,22^ Because of this, Bonnot *et al*^2^ incorporate an abdominal ultrasound scan into their diagnostic ‘work-up’ algorithm for IEMs causing a schizophrenia-like illness.^2^

A patient with splenomegaly (especially in the absence of liver disease) with a co-existent neurodegenerative or psychiatric disorder is strongly suggestive of NP-C^4^ and should be appropriately investigated. A history of paediatric liver disease in such patients should also raise clinical suspicion.

## Investigation and diagnosis

Rapid advancements in gene sequencing and liquid chromatography/tandem mass spectrometry (LC-MS/MS) have led to significant change in the available approaches to diagnosing NP-C, with both easier and more affordable tests available or in development.^28^

Bonnot *et al*^2^ suggest an algorithm for the work-up of a patient with a schizophrenia-like illness and a possible IEM. They suggest that with initial suspicion, a clinical and ophthalmological assessment and a cerebral magnetic resonance imaging (MRI) scan should be performed. Subsequent investigations should be performed based on these findings, with an abdominal ultrasound scan to identify hepatosplenomegaly if NP-C is considered. If this is positive, then disease-specific NP-C tests can be performed.^2^ However, this pragmatic approach has some limitations: not all patients with NP-C have hepatosplenomegaly,^4,5,22^ and with easier plasma diagnostic tests available these should be performed earlier in the diagnostic process.

Historically, the diagnosis of NP-C was made histopathologically, by both cholesterol esterification studies and filipin staining of cultured skin fibroblasts,^4^ with most patients receiving a combination of different tests performed prior to this good, but costly and difficult, definitive investigation. These tests may have included: chitotriosidase measurements, white cell enzyme studies to exclude other lysosomal storage disorders, and fluorescent and electron microscopy of both bone marrow aspirate and liver biopsy specimens.^28^ Because of the difficulties with the filipin staining test, the most widely performed and accessible definitive diagnostic test is now the sequencing of the *NPC1* and *NPC2* genes. Next-generation sequencers make this far easier to perform, especially if the genes concerned are included on a multi-gene panel appropriate for patients presenting with a certain disease phenotype – such as neonatal cholestatic jaundice.^29^ But this approach is not without some limitations either. In 10% of patients only a single pathogenic mutation can be identified, and in some patients new mutations of uncertain clinical significance may be identified.

More recently, highly specific and sensitive oxidative cholesterol metabolites for NP-C have been identified.^30^ This ‘oxysterol test’ can be performed on a plasma sample and is now used as the first-line diagnostic test with subsequent genetic confirmation at one of the principal UK reference laboratories for lysosomal storage disorders. Although it has a positive predictive value of > 97% in an NP-C enriched population such as infants with cholestatic jaundice,^28^ its accuracy as a screen in broader populations is still being clarified. With the recent advances in LC-MS/MS, other candidate metabolites for diagnostic tests are being identified, with several in the pipeline. These are likely to be available in the near future as cheaper and widely accessible plasma or urine diagnostic tests.^28^

## Treatment options

### Disease-specific treatments

Miglustat, a small iminosugar molecule, is licensed in the European Union for the treatment of the progressive neurological manifestations of NP-C in both adults and children.^4^ It reduces the accumulation of the downstream toxic metabolites, glycosphingolipids (GSL), by competitively inhibiting the first step in their synthesis.^31^ It has been shown to stabilise certain key neurological manifestations in a randomised controlled trial (RCT), a retrospective cohort study and in clinical experience.^4,32,33^ However, in adults it may take a year or longer to identify a discernible clinical benefit.^4^

Multiple other therapies are currently under clinical investigation for NP-C, of which two studies are at the Phase 2b/3 pivotal trial stage:

- arimoclomol, a small molecule that induces the heat shock protein response – a normal cellular stress response^34^
- cyclodextrins, ring-like sugar molecules that reduce lipid storage and in animal models have both substantially reduced the burden of disease and greatly prolonged lifespan.^35,36^

### Symptomatic treatments

Complex neuropsychiatric diseases have a profound effect on the patient, their family and carers. Consideration of the patient's nutritional status, swallow safety and toileting/bowel function, as well as their mobility and safety, is important, with a multidisciplinary team involved and access to appropriate agencies as needed. Timely discussions around issues of capacity, care and end-of-life planning are also necessary.

#### Cognitive impairment

Appropriate support services should be involved. Although miglustat may stabilise the cognitive decline, there is no evidence that cognitive-enhancing drugs such as cholinesterase inhibitors have a beneficial role.^4^

#### Psychiatric illness

Psychosis usually responds to antipsychotic medications, but some NP-C patients are resistant to treatment or even show (paradoxical) worsening with the initiation of drug therapy (a useful diagnostic red flag in unidentified NP-C). Atypical antipsychotics should be used and frequent neurological assessments performed to identify worsening of any pre-existing dystonia. If it occurs, dose reduction or an alternative antipsychotic may be used, supplemented if necessary with sodium valproate.^4^ Depression typically responds well to SSRIs,^37^ and in some patients, when effectively treated, this leads to improvements not only in their mood but also their cognition and neurological disease. Bipolar disorder in NP-C has responded to mood stabilisers such as sodium valproate and catatonia has been treated successfully with ECT.^4,18^ Sleep disturbance in NP-C may manifest as sleep inversion, narcolepsy or obstructive sleep apnoea and can be treated with melatonin and continuous positive airway pressure ventilation (CPAP).^4^

#### Neurological disease

Patterson *et al*^4^ have published recommended treatment strategies for a range of different NP-C neurological complications.

## Prognosis

Accurate prognostic predictions in NP-C are difficult. There is poor genotype-phenotype correlation in disease course, with affected siblings not infrequently following different disease trajectories. The extent and severity of visceral disease offers little insight into the severity of later neurological disease, an additional challenge when counselling parents of a newly diagnosed infant with liver disease.

The most useful prognostic indicator is the age at neuropsychiatric disease onset.

## Summary

The largest subgroup of NP-C patients is likely to be an undiagnosed/misdiagnosed adult population with a neuropsychiatric disease. Consequently, NP-C highlights the need for continual diagnostic review in patients with psychosis, especially if there is coexistent cognitive decline and/or ataxia.
